# Supplementary material for: Usability of EU-TIRADS in the Diagnostics of Hürthle Cell Thyroid Nodules with Equivocal Cytology
Source: J Clin Med. 2020 Oct 24;9(11):3410. doi: 10.3390/jcm9113410 (PMC7690849; doi:10.3390/jcm9113410)
Supplement: Supplementary file 1 [file jcm-09-03410-s001.pdf]

**Table S1.** Comparison of the incidence of sonographic features in HC nodules classified into particular equivocal categories of BSRTC in relation to the histopathological outcome: benign lesion vs thyroid malignancy.

| Sonographic feature | HC nodules–category of BSRTC | | | | | | | | |
|---|---|---|---|---|---|---|---|---|---|
| | III | | | IV | | | V | | |
| | Ben. (45) No/% | Mal. (8) No/% | *p* | Ben. (83) No/% | Mal. (14) No/% | *p* | Ben. (5) No/% | Mal. (7) No/% | *p* |
| marked hypoechogenicity | 0/0 | 1/12.5 | NS | 6/7.2 | 5/35.7 | <0.005 | 0/0 | 1/14.3 | NS |
| hypoechogenicity | 28/62.2 | 6/75.0 | NS | 63/75.9 | 11/78.6 | NS | 4/80.0 | 7/100.0 | NS |
| solid echostructure | 39/86.7 | 6/75.0 | NS | 78/94.0 | 14/100.0 | NS | 4/80.0 | 5/71.4 | NS |
| more solid than cystic | 44/97.8 | 7/87.5 | NS | 83/100.0 | 14/100.0 | - | 4/80.0 | 7/100.0 | NS |
| spongiform echostructure | 1/2.2 | 0/0/0 | NS | 0/0/0 | 0/0/0 | NS | 1/20.0 | 0/0/0 | NS |
| suspicious shape | 4/8.9 | 1/12.5 | NS | 11/13.3 | 4/28.6 | NS | 0/0 | 2/28.6 | NS |
| irregular margins | 0/0 | 2/25.0 | <0.05 | 3/3.6 | 0/0 | NS | 0/0 | 3/42.9 | NS |
| microcalcifications | 1/2.2 | 2/25.0 | NS | 4/4.8 | 1/7.1 | NS | 0/0 | 2/28.6 | NS |
| macrocalcifications | 2/4.4 | 1/12.5 | NS | 1/1.2 | 1/7.1 | NS | 0/0 | 1/14.3 | NS |
| rim calcifications | 0/0 | 0/0 | - | 3/3.6 | 2/14.3 | NS | 0/0 | 0/0 | - |
| pathological vascularization | 9/20.0 | 1/12.5 | NS | 21/25.3 | 5/35.7 | NS | 0/0 | 3/42.9 | NS |

*-none of the nodules of both groups did not present a pure cystic echostructure. Ben.–benign lesion in histopathological outcome. Mal. – thyroid malignancy in histopathological outcome.

**Table S2.** Comparison of the incidence of sonographic features in non-HC classified into particular equivocal categories of BSRTC in relation to the histopathological outcome: benign lesion vs thyroid malignancy.

| Sonographic feature | non-HC nodules–category of BSRTC | | | | | | | | |
|---|---|---|---|---|---|---|---|---|---|
| | III | | | IV | | | V | | |
| | Ben. (249) No/% | Mal. (27) No/% | *p* | Ben. (70) No/% | Mal. (5) No/% | *p* | Ben. (5) No/% | Mal. (27) No/% | *p* |
| marked hypoechogenicity | 20/8.0 | 6/22.2 | NS | 8/11.4 | 1/20.0 | NS | 0/0.0 | 7/25.9 | NS |
| hypoechogenicity | 183/73.5 | 23/85.2 | NS | 48/68.6 | 4/80.0 | NS | 2/40.0 | 22/81.5 | NS |
| solid echostructure | 185/74.3 | 23/85.2 | NS | 54/77.1 | 5/100.0 | NS | 4/80.0 | 24/88.9 | NS |
| more solid than cystic | 227/91.2 | 27/100.0 | NS | 62/88.6 | 5/100.0 | NS | 5/100.0 | 26/96.3 | NS |
| spongiform echostructure | 10/4.0 | 0/0.0 | NS | 1/1.4 | 0/0.0 | NS | 0/0.0 | 0/0.0 | - |
| suspicious shape | 24/9.6 | 5/18.5 | NS | 10/14.3 | 1/20.0 | NS | 0/0.0 | 5/18.5 | NS |
| irregular margins | 11/4.4 | 4/14.8 | NS | 3/4.3 | 1/20.0 | NS | 0/0.0 | 13/48.1 | NS |
| microcalcifications | 7/2.8 | 5/18.5 | <0.0001NS | 5/7.1 | 1/20.0 | NS | 0/0.0 | 3/11.1 | NS |
| macrocalcifications | 19/7.6 | 3/11.1 | NS | 4/5.7 | 1/20.0 | NS | 0/0.0 | 4/14.8 | NS |
| rim calcifications | 6/2.4 | 1/3.7 | NS | 4/5.7 | 1/20.0 | NS | 0/0.0 | 1/3.7 | NS |
| pathological vascularization | 50/20.1 | 9/33.3 | NS | 23/32.9 | 1/20.0 | NS | 4/80.0 | 6/22.2 | <0.05 |

*-none of the nodules of both groups did not present a pure cystic echostructure. Ben.–benign lesion in histopathological outcome. Mal. – thyroid malignancy in histopathological outcome.

**Table S3.** Change from FNA-ROM of a nodule in relation to its EU-TIRADS category. (EU-TIRADS category corresponding to the lack of nodules has been omitted).

| | Category of TIRADS | Category of BSRTC | | | | | | | | |
|---|---|---|---|---|---|---|---|---|---|---|
| | | III | | | IV | | | V | | |
| | | ben./mal. nodules | T-RoM (%) | FNA-RoM vs T-RoM P | ben./mal. nodules | T-RoM (%) | FNA-RoM vs T-RoM P | ben./mal. nodules | T-RoM (%) | FNA-RoM vs T-RoM P |
| HC nodules | | FNA-RoM: 15.1% | | | FNA-RoM: 14.4% | | | FNA-RoM: 58.3% | | |
| | benign | 1/0 | 0.0 | NS | - | - | - | 1/0 | 0.0 | NS |
| | low risk | 14/2 | 12.5 | NS | 16/3 | 15.8 | NS | 1/0 | 0.0 | NS |
| | intermediate risk | 25/3 | 10.7 | NS | 44/4 | 8.3 | NS | 3/4 | 57.1 | NS |
| | high risk | 5/3 | 37.5 | NS | 23/7 | 23.3 | NS | 0/3 | 100.0 | NS |
| non-HC nodules | | FNA-RoM: 9.8% | | | FNA-RoM: 7.1% | | | FNA-RoM: 84.4% | | |
| | benign | 9/0 | 0.0 | NS | 1/0 | 0.0 | NS | - | - | NS |
| | low risk | 64/2 | 3.0 | NS | 13/0 | 0.0 | NS | 3/4 | 57.1 | NS |
| | intermediate risk | 127/10 | 7.3 | NS | 29/2 | 6.5 | NS | 2/5 | 71.4 | NS |
| | high risk | 49/15 | 23.4 | 0.005 | 22/3 | 12.0 | NS | 0/18 | 100.0 | NS |
